# Correspondence

**Published:** 1885-04

**Authors:** Flavel S. Thomas


### CORRESPONDENCE.

*To the Editors of the* JOURNAL OF COMPARATIVE MEDICINE AND SURGERY:

Why do the schools confer so many different degrees when they all mean the same thing? To the uniniated J. Blank, V.S., D.V.S., D.V.M., must seem to be a very learned man, but we know that it would mean no more than simply V.S.

In am sorry that some of our schools are borrowing the "Doctor" from the older degrees; it does not give additional worth to the degree. D.V.S. which is conferred upon the completion of two courses of lectures, cannot be worth as much as V.S., Montreal, which is only conferred upon the completion of three courses of lectures.

The doctorate degrees are worthy, old and honorable degrees; now it seems to me that the idea is to steal some of the honor which these degrees have gained by hard work—centuries of hard work—and to tack it on to a degree which is just born, which can scarcely stand alone, as yet; and perhaps that is the trouble—it cannot easily stand alone, so it seizes the cane and wig from one of the old men (doctorates) and proclaims that it is an old man too; but it does not deceive the world; it will not long at any rate.

Let us cling to the first, to the original, to the most expressive, to the most valuable degree, V.S. If we are ashamed of V.S., let us not deck ourselves out in borrowed plumage—borrowed or stolen honors—but let us cling to V.S., and *make it* honorable.

Then, again, M.R.C.V.S. is an absurdity, and H.F.R.C.V.S. is worse yet.

Why not add the whole alphabet? This only means V.S., and the V.S. could be found somewhere in the alphabet, the same as it can in H.F.R.C.V.S., and about as easily. I do not object to a string of letters after a man's name when every two or even three indicate the completion of a special course of study, but when a man uses three, five or six, the meaning of which might be expressed by two, I say it is absurd.

I greatly respect J. Blank, Ph.B., M.D., V.S., but when I read J. Blank, M.R.C V.S., or J. Blank, H.F.R.C.V.S., I smile.

FLAVEL S. THOMAS, A.M., V.S.

